# Monkeypox virus clade IIb isolate exhibits reduced virulence relative to clade IIa isolates in multiple murine models

**DOI:** 10.1128/jvi.00247-26

**Published:** 2026-07-15

**Authors:** Stephanie V. Trefry, Santiago Vidal-Freire, Mayanka Awasthi, Abel D. Ordonez, Brett P. Eaton, Randy J. Hart, Christy N. Raney, Amy L. Cregger, Chase A. Gonzales, Robert N. Enamorado, Nelson A. Martinez, Deborah S. Gohegan, Matthew G. Lackemeyer, Tinoush Moulaei, Natasza E. Ziółkowska, Jeremy J. Bearss, Scott J. Goebel, Gustavo Palacios, Sina Bavari, Farooq Nasar

**Affiliations:** 1 Tonix Pharmaceuticals 466315, Frederick, Maryland, USA; 2 Department of Microbiology, Icahn School of Medicine at Mount Sinai 479914, New York, New York, USA; 3 Global Health Emerging Pathogens Institute, Icahn School of Medicine at Mount Sinai 5925, New York, New York, USA; Northwestern University Feinberg School of Medicine, Chicago, Illinois, USA

**Keywords:** mouse models, monkeypox virus, virulence

## Abstract

Monkeypox virus (MPXV) is the causative agent of mpox disease in humans. The virus comprises two clades, Central African clade I and West African clade II, with case fatality rates of ~11 and ~4%, respectively. Since the discovery of mpox disease in 1970, the virus has been restricted to Africa. However, in 2022, a previously unrecognized subclade IIb caused the largest global outbreak of mpox disease with a case fatality rate of ~0.2%. The difference in virulence of MPXV subclades in human infection warrants further investigation; however, one critical limitation is the lack of susceptible small animal models. In this study, we investigated the susceptibility of four murine models, including CAST-EiJ and three immunocompromised models (C57BL/6 *Ifnar*^−/−^, C57BL/6 *Ifngr*^−/−^, and C57BL/6 *Ifnar*^−/−^/*Ifngr*^−/−^) to MPXV clade IIa (WR 7-61 and US-2003) and IIb (MA-2022) isolates. All four mouse models were susceptible to clade IIa infection, leading to severe disease marked by decreased body temperature, weight loss, and lethality. In contrast, clade IIb infection produced minimal to mild disease at similar doses in all four murine models. The clade IIb isolate produced severe disease (40% lethality) at only the highest dose (8.0 log_10_ PFU) in the most susceptible immunocompromised mouse model, C57BL/6 *Ifnar*^−/−^/*Ifngr*^−/−^. This is the first demonstration of lethal disease with clade IIb in a murine model. In addition, these data show that clade IIa is ~100 to 100,000-fold more virulent than clade IIb and provides three additional murine models for investigating MPXV infection and pathogenesis.

**IMPORTANCE:**

Mpox is an emerging human disease caused by four distinct MPXV subclades (Ia, Ib, IIa, and IIb). Despite genetic similarities, the case fatality rate varies considerably between the subclades: Ia (~11%), Ib and IIa (~4%), and IIb (~0.2%). Since 2022, multiple mpox outbreaks have occurred due to previously unrecognized subclades, leading to the declaration of two public health emergencies by the World Health Organization. This unprecedented global spread, coupled with the variation in the severity of human disease, underscores the importance of research into the pathogenesis of emerging MPXV subclades. However, a critical limitation is the lack of suitable small animal models. This study identifies three additional murine models susceptible to MPXV clade II infection and demonstrates significant virulence differences between clades IIa and IIb. These models will enable a rapid characterization of previously unrecognized subclades and will facilitate countermeasure development.

## INTRODUCTION

Monkeypox virus (MPXV) is a member of the genus *Orthopoxvirus* in the family *Poxviridae*. The genome comprises a linear double-stranded DNA molecule ~197 to 200 kilobase pairs (kbp) in length with covalently enclosed ends encoding ~190 proteins (1–4). Mpox is a zoonotic disease endemic in parts of Central and West Africa (2, 5). The natural cycle required for maintenance of MPXV in nature is unknown; however, studies suggest that squirrels, giant pouched rats, African dormice, and non-human primates (NHPs) can serve as sylvatic reservoir hosts (6–16). MPXV was first discovered in 1958 in Denmark in a colony of cynomolgus macaques exhibiting a disease similar to smallpox (17). In the subsequent decade, the virus was isolated from multiple outbreaks in NHP colonies in the USA and Europe following the importation of infected animals from Africa (18–22).

The first human case of mpox disease was reported in a young boy unvaccinated against smallpox in 1970 in Zaire (now the Democratic Republic of the Congo) (23). In the following five decades, sporadic outbreaks were reported in West and Central Africa (2, 5). Human transmission of MPXV occurs through dermal or respiratory routes and can cause severe disease characterized by fever, headache, fatigue, rash, lymphadenopathy, cutaneous lesions, myocarditis, encephalitis, and death (2, 24).

MPXV is divided into two clades, clades I (Central African or Congo Basin) and II (West African), based on phylogenetics, geography, and the severity of human disease (25–28). Since the first reported human case of mpox disease, clade I isolates have accounted for a vast majority of the human cases. Sporadic outbreaks between 1970 and 2020 produced ~30,000 confirmed or suspected cases with a case fatality rate of up to ~11% (5). In contrast, only 200 cases were reported for clade II during the same period with a case fatality rate of ~4% (5). For more than half a century, human mpox outbreaks have been confined to Africa. However, in 2022, the largest mpox outbreak occurred and rapidly spread throughout the globe (29, 30). The genetic sequencing of the 2022 mpox isolates revealed that the outbreak was caused by a previously unrecognized subclade IIb. Phylogenetic analysis of the outbreak isolates demonstrated a close relationship to endemic isolates from Nigeria that have been circulating since 1971 and were responsible for the travel acquired cases exported to Europe and Asia in the 2017 outbreak (31–37). To date, the outbreak has produced ~100,000 confirmed cases across 122 countries, with 220 reported deaths and a case fatality rate of ~0.2% (38, 39). In contrast to previous outbreaks, 96% of infections were reported in men who have sex with men (MSM) (38, 39). This is the first recorded instance of mpox disease through sexual transmission and highlights the potential of MPXV to cause global outbreaks.

Following the 2022 global outbreak, multiple outbreaks have occurred due to yet another previously unrecognized subclade, clade Ib (40–44). Consequently, MPXV now comprises two main clades, with each further subdivided into subclades: Ia, Ib, IIa, and IIb. The human case fatality rates among the four subclades vary considerably, with rates of ~11% (Ia), ~4% (Ib and IIa), and ~0.2% (IIb). The differences in virulence between MPXV clades in humans warrant further investigation; however, a critical impediment is the scarcity of small animal models. Comprehensive studies investigating the susceptibility of laboratory mouse strains to MPXV infection have been conducted previously (6, 39). Only four mouse strains ( suckling white mice, SCID-BALB/c, C57BL/6 *stat1^−/−^*, and CAST/EiJ) were shown to be susceptible to MPXV infection (6, 45–48). Of these models, C57BL/6 *stat1^−/−^* and CAST/EiJ have been utilized to investigate viral pathogenesis, as well as countermeasure development (48–50). In addition, the majority of MPXV animal studies have utilized clade I isolates (6–16, 26, 45–60). Consequently, few publications have compared the pathogenesis of clade IIa and IIb isolates (6, 45). In this study, we investigated the virulence differences of two MPXV clade IIa (WR 7-61 and US-2003) and one clade IIb (MA-2022) isolates in CAST/EiJ mice, as well as three immunocompromised mouse strains (C57BL/6 *Ifnar^−/−^*, C57BL/6 *Ifngr^−/−^*, and C57BL/6 *Ifnar*^−/−^/*Ifngr^−/−^*).

## RESULTS

### *In silico* and *in vitro* characterization of clade II challenge stocks

**Fig 1:**
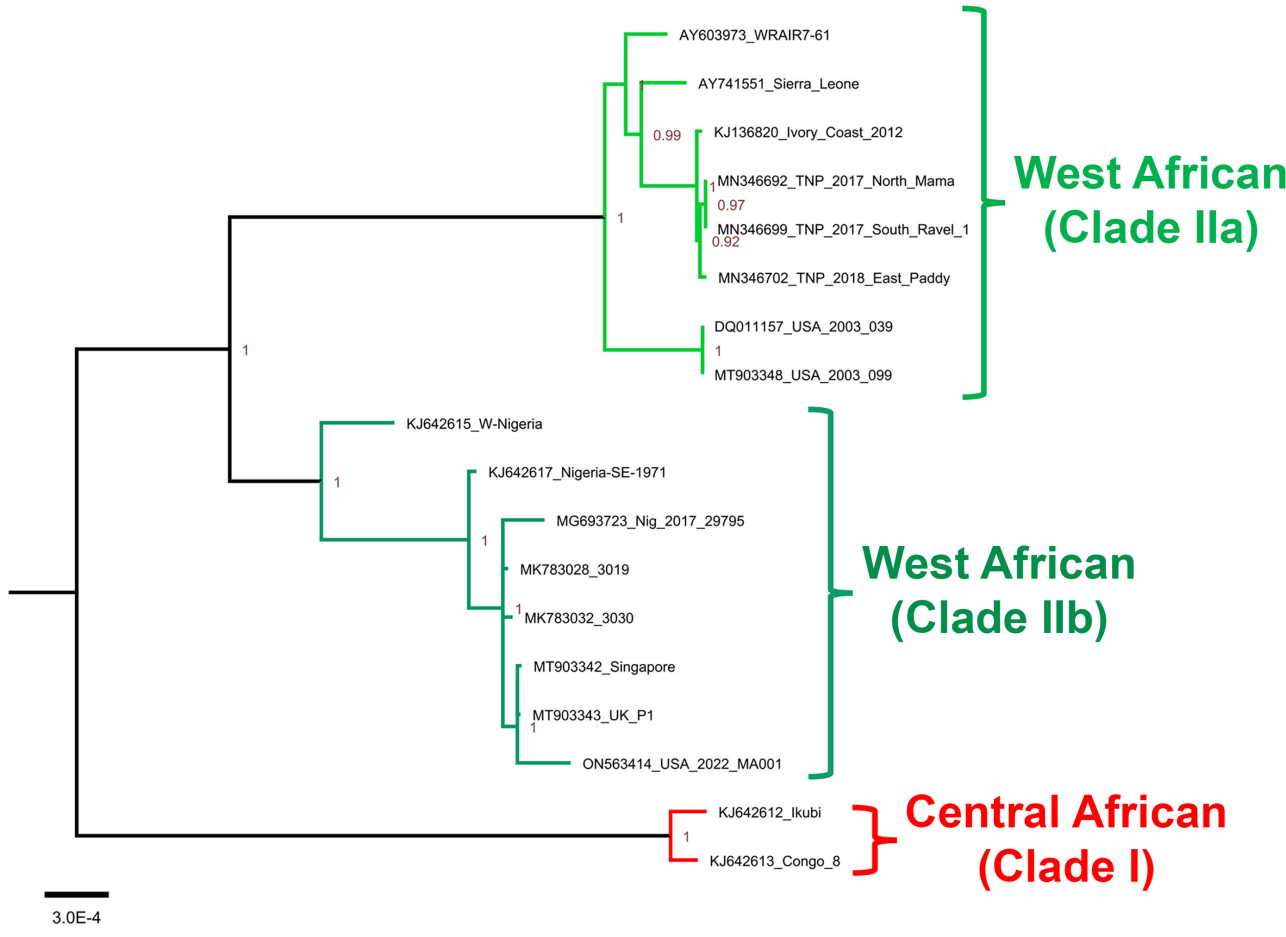
Phylogenetic relationship of MPVX isolates. The maximum likelihood tree based on the whole genome is shown with Central African (clade I—red), West African clade IIa (light green), and West African clade IIb (dark green).

To assess the evolutionary relatedness of MPXV, representative sequences from clades I and II were downloaded, aligned, and used to generate a maximum-likelihood phylogenetic tree (Fig. 1). As expected, two main clades were observed with two branches consisting of clade I Central African isolates and clade II West African isolates. Clade II further branched into clades IIa and IIb, with clade IIa comprising the oldest West African MPXV isolates WR 7-61 (1962) and Sierra Leone (1970). In contrast, clade IIb comprised endemic isolates from Nigeria and travel-acquired MPXV infections exported abroad. The MA-2022 isolate was placed within clade IIb, sister to isolates from Singapore and the United Kingdom.

Given the renewed interest in employing well-characterized representative isolates in validated animal models, available sequence data from clade IIa and IIb MPXV isolates was analyzed to characterize the variations in selected challenge stocks (61). The analysis identified gene variations across MPXV clades as well as clade-specific differences in gene length, truncation, and deletions (Table 1). Additionally, gene polymorphisms were documented that distinguished the selected challenge stocks in this study from their respective clades (Tables S1a and b). The phenotypic significance or potential correlations of these variations are beyond the scope of this work.

**TABLE 1:** Gene variations observed among MPXV clades^*a*^

Orthopox gene (OPG)	VACV-COP ortholog	CPXV-Bri ortholog	MPXV clade Ia ortholog	MPXV clade IIa ortholog	MPXV clade IIb ortholog	CPXV accession no.	CPXV full-length (aa)	Variation relative to CPXV	MPXV clade Ia length (aa)	MPXV clade IIa length (aa)	MPXV clade IIb length (aa)	Protein domain, structure, localization, function
Genes missing in one or more MPXV clades												
OPG016	No homolog	CPXV018	NP	N3R	NP	CAD90591.1	178	Only present (truncated) in Clade IIa	0	176	0	MHC class I homolog, secreted, blocks NKG2D receptor, OMCP
OPG017	No homolog	CPXV019	NP	N2R	NP	CAD90592.1	833	Only present (truncated) in Clade IIa	0	73	0	ANK and PRANC domains, binds to Cullin 2, inhibits NF-kB activation
OPG005	B22R/C16L	CPXV009	NP	N1R	NP	CAD90589.1	153	Full-length only present in Clade IIa	0	153	0	Bcl-2 domain, host range factor
OPG032	C3L	CPXV034	D14L	NP	NP	CAA64102.1	259	Only present (truncated) in Clade Ia	216	0	0	Complement control protein (CCP), prevents complement activation, secreted
OPG033	C2L	CPXV035	Truncated	NP	Truncated	CAA64103.1	512	Present (truncated) in Clade Ia and IIb	105	0	126	Ubiquitin Targeting Immune evasion, virulence factor
OPG195	B9R	CPXV203	B10R	Truncated	B10R	CAD90734.1	221	Full-length only present in Ia and IIb	221	72	221	PIE, ER localized, retains MHC I in ER, prevents antigen presentation
OPG201	B16R	CPXV209	B14R, Truncated	NP	Truncated	CAD90740.1	326	Present (truncated) in Clade Ia, IIb	210	0	180	three immunoglobulin domains, IL-1 receptor homolog, blocks IL-1b, prevents fever in mice
OPG202	B17L	CPXV210	B15L, Truncated	NP	NP	CAD90741.1	340	Only present (truncated) in Clade Ia	78	0	0	Distant homolog of A51 protein
Genes with variations in length in some MXPV clades												
OPG029	C6L	CPXV030	D11L	D11L	D11L	CAA64099.1	156	Polymorphism, STR repeat	153	158	155	Bcl-2 domain, inhibits IFN Type-I production and signaling, targets HDAC5 for degradation
OPG037	M1L	CPXV039	O1L	O1L	O1L	CAA64107.1	474	Polymorphism, STR repeat	441	445	442	ANK, no PRANC domain, inhibits intrinsic apoptosis at the level of the apoptosome
OPG153	A26L	CPXV159	A28L	A28L	A28L	CAD90694.1	518	Polymorphism, STR repeat	512-513	509-510	499-511	P4c precursor; A28L; major component of IMV surface tubules; p4c
OPG204	B19R	CPXV212	B16R	B16R	B16R	CAD90743.1	351	Polymorphism, STR repeat	350	354	356	three immunoglobulin domains, IFN type-1 decoy receptor, secreted, attaches to GAGs
OPG208	K2L	CPXV217	B19R	B19R	B19R	CAD90746.1	375	Polymorphism, STR repeat	389-392	524-542	449-497	Serpin 1,2,3; SPI-1; B19R; serine protease inhibitor-like, SPI-1; apoptosis inhibition
Genes with variations in length in all MXPV clades												
OPG020	C10L	CPXV022	Truncated	Truncated	Truncated	CAD90594.1	331	Truncated in MPXV	83	67	37	N-term prolyl hydroxylase fold, C-term IL-1 receptor antagonist, binds Ku
OPG027	C7L	CPXV029	Truncated	Truncated	Truncated	CAA64098.1	150	Truncated in MPXV	64	105	72	Host range, inhibits type I IFN production and signaling
OPG041	C3L	CPXV043	Truncated	Truncated	Truncated	CAA64111.1	88	Truncated in MPXV	43	43	43	Mimic of eIF2a, pseudo-substrate for PKR, PKR inhibitor, IFN resistance

^*a*^ OPG = Orthopox gene, VACV-COP = Vaccinia virus Copenhagen isolate, CPXV-Bri = Cowpox virus Brighton Red isolate, MPXV = Monkeypox virus, CPXV = Cowpox virus, aa = amino acid, NP = not present.

**Fig 2:**
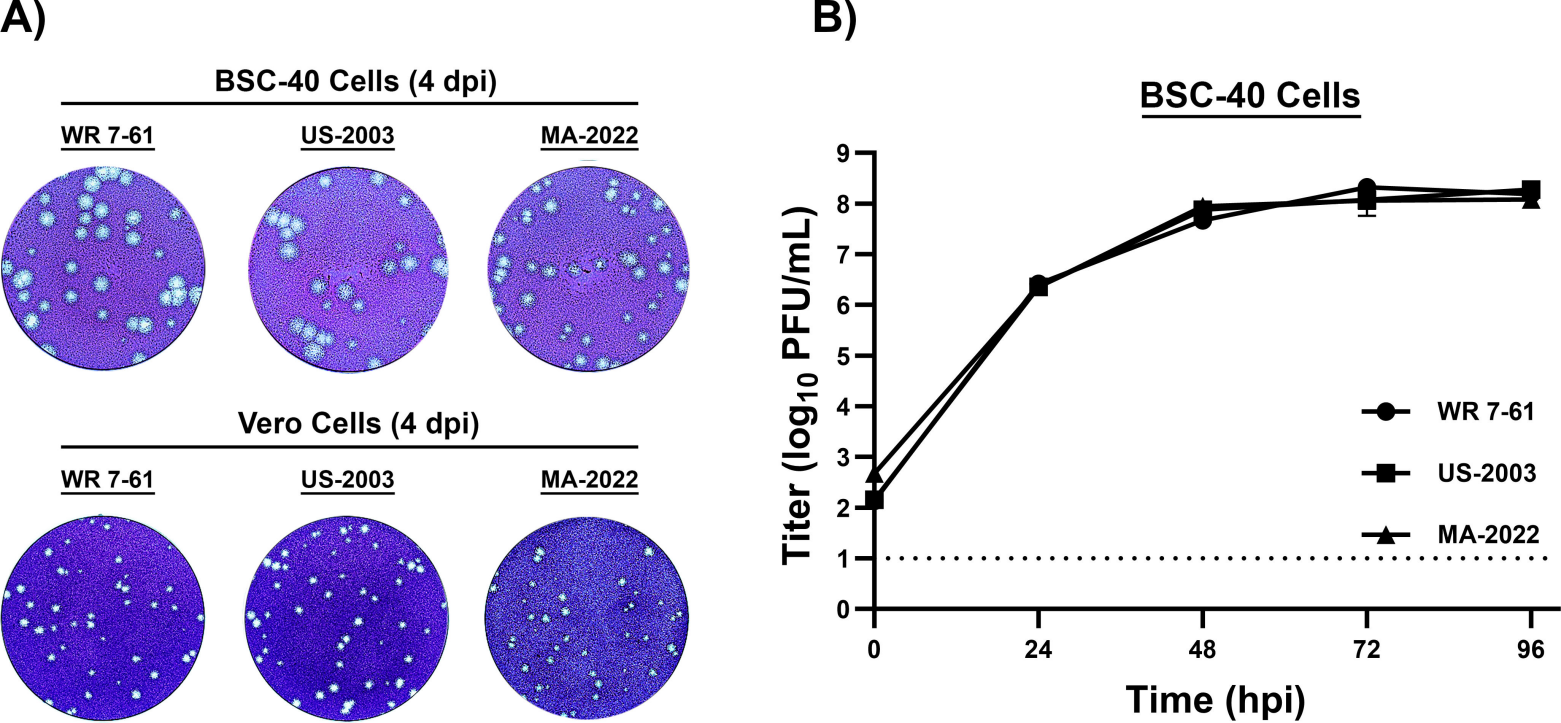
Plaque phenotype and multi-step growth kinetics of MPXV clade IIa isolates (WR 7-61 and US-2003) and IIb isolate (MA-2022). Plaque phenotype of MPXV isolates WR 7-61, US-2003, and MA-2022 was investigated in BSC-40 and Vero cells (**A**). Representative wells from six-well plates at 4 dpi are shown. Multi-step growth kinetics of MPXV isolates in BSC-40 cells at an MOI of 0.01 (**B**). Samples were collected at 0, 24, 48, 72, and 96 hpi. Triplicate six-wells were sampled at each time point, followed by three cycles of freeze/thaw/sonication, and titration on BSC-40 cells. The limit of detection for infectious virus (1.0 log_10_ PFU/mL) is indicated with black dashed lines.

The plaque phenotype of all three isolates was investigated in Vero and BSC-40 cells (Fig. 1A). By 4 dpi, all three isolates produced plaques ~1 to 2 and ~2 to 3 mm in diameter on Vero and BSC-40 cells, respectively. In both cell lines, WR 7-61 and US-2003 isolates produced a predominantly uniform plaque phenotype, whereas MA-2022 isolate yielded a mixed plaque phenotype. The isolates were further analyzed by multi-step growth kinetics in BSC-40 cells. All three isolates displayed similar replication kinetics with a ~10,000-fold increase in titer by 24 hpi and achieved a peak titer of ~8.0 log_10_ PFU/mL at 72 hpi (Fig. 2B).

### Virulence difference between MPXV clade IIa (WR 7-61) and IIb (MA-2022) isolates in 7- to 8-week-old CAST/EiJ mice via the intranasal route

**Fig 3:**
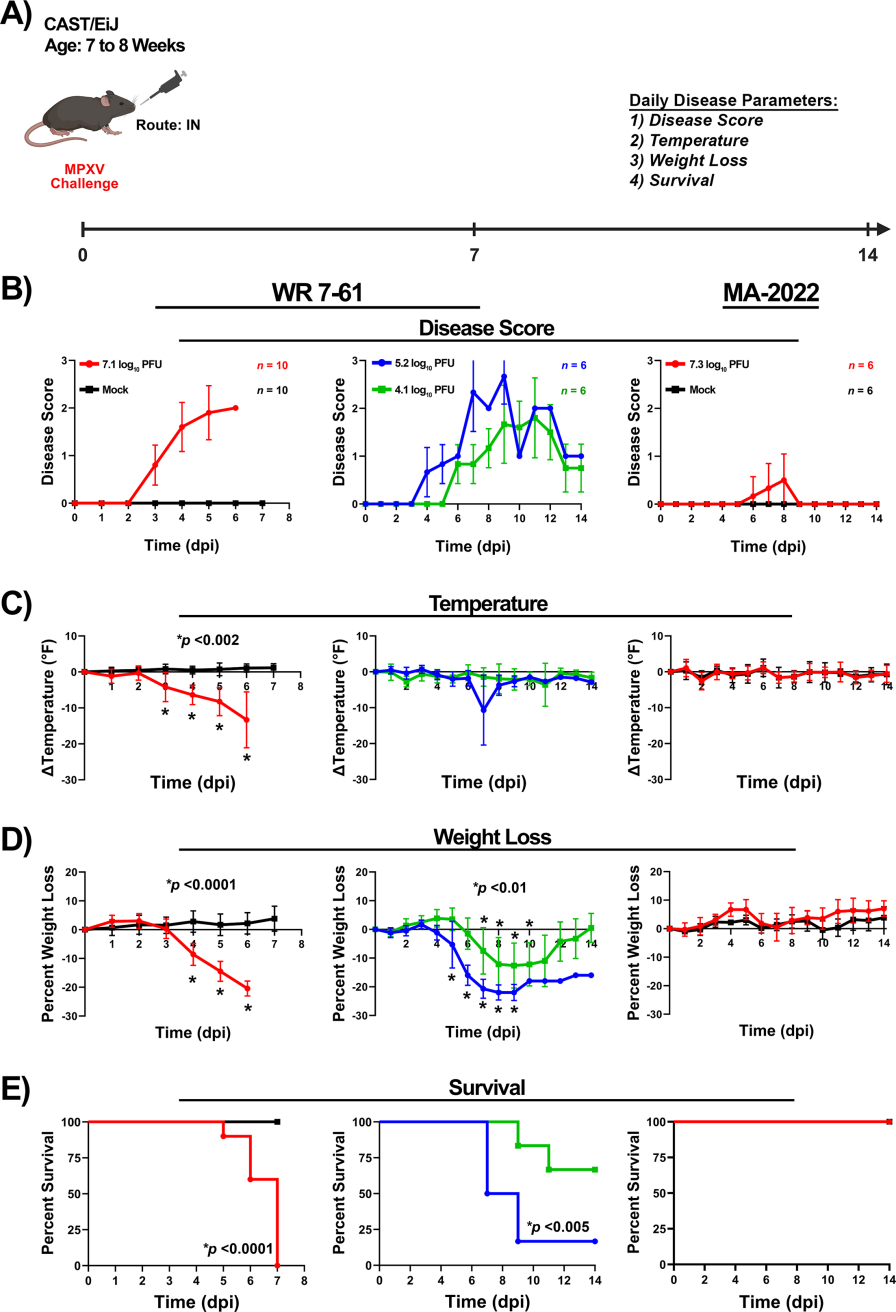
Infection of 7- to 8-week old CAST/EiJ mice with MPXV clade IIa (WR 7-61) and IIb (MA-2022) isolates via the intranasal (IN) route. Mice were infected with WR 7-61 at 7.0, 5.0, or 4.0 log_10_ PFU per mouse and with MA-2022 at 7.0 log_10_ PFU per mouse (**A**). Number of mice per group (*n*) ranged from 6 to 10 and is indicated for respective dose group (**B**). Following infection, mice were monitored for disease score (**B**), body temperature (**C**), weight loss (**D**), and survival (**E**). The target dose for each virus was verified by plaque assay, and values are shown (**A**). *P-values comparing MPXV isolates with the mock group are provided in each graph.

Cohorts of 6 to 10 mice were infected with WR 7-61 (7.0, 5.0, or 4.0 log_10_ PFU/mouse), or MA-2022 (7.0 log_10_ PFU/mouse), or were mock-treated (Fig. 3A). Following infection, mice were monitored daily for disease score, body temperature, weight loss, and survival for 14 days. Severe disease was observed in mice infected with the WR 7-61 isolate in a dose-dependent manner. Mice infected at 7.0 log_10_ PFU exhibited increased disease score by 3 dpi with a peak average score of ~2 at 6 dpi (Fig. 3B). Body temperature and weight declined beginning at 3 and 4 dpi, with peak average reduction of ~−13 °F and ~−20% at 6 dpi, respectively (Fig. 3C and D). All mice met euthanasia criteria by 7 dpi (Fig. 3E).

Mice infected with WR 7-61 at 5.0 and 4.0 log_10_ PFU/mouse exhibited increased disease scores between 4 and 6 dpi, with peak average scores of ~2.7 and ~1.8 at 9 and 11 dpi, respectively (Fig. 3B). Alterations in body temperature were not observed in either dose group, except at 7 dpi in the 5.0 log_10_ PFU group, with mice exhibiting peak average temperature decline of ~−11 °F (Fig. 3C). Body weight declined in both dose groups between 5 and 6 dpi, with a peak average reduction of ~−22% (5.0 log_10_ PFU) and ~−13% (4.0 log_10_ PFU) (Fig. 3D). Eighty-three and thirty-three percent of the mice in 5.0 and 4.0 log_10_ PFU groups met the euthanasia criteria, respectively (Fig. 3E).

In contrast to WR 7-61-infected mice, minimal disease was observed in mice infected with MA-2022. Disease scores increased between 6 and 8 dpi, with peak score of ~0.5 (Fig. 3B). Minimal or no alterations were observed in body temperature or weight, and all mice survived the 14-day study (Fig. 3C through E).

### Virulence difference between MPXV clade IIa (WR 7-61) and IIb (MA-2022) isolates in four- to five-month-old CAST/EiJ mice via the intranasal route

**Fig 4:**
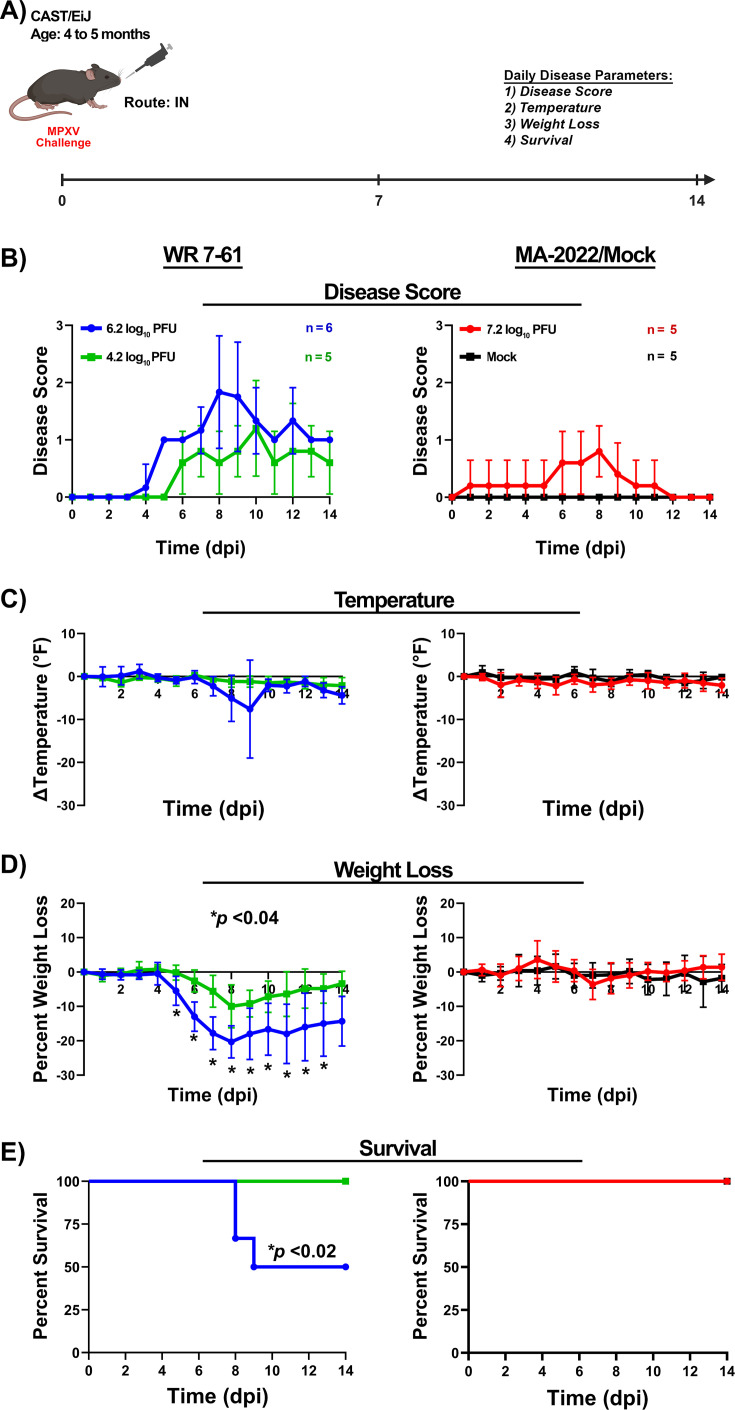
Infection of 4- to 5-month old CAST/EiJ mice with MPXV clade IIa (WR 7-61) and IIb (MA-2022) isolates via the intranasal (IN) route. Mice were infected with WR 7-61 at 6.0 or 4.0 log_10_ PFU per mouse and with MA-2022 at 7.0 log_10_ PFU per mouse (**A**). Number of mice per group (*n*) ranged from 5 to 6 and is indicated for respective dose group (**B**). Following infection, mice were monitored for disease score (**B**), body temperature (**C**), weight loss (**D**), and survival (**E**). The target dose for each virus was verified via the plaque assay, and values are provided (**A**). *P-values comparing MPXV isolates with the mock group are provided in each graph.

Cohorts of five to six mice were infected with WR 7-61 (6.0 or 4.0 log_10_ PFU/mouse), or MA-2022 (7.0 log_10_ PFU/mouse), or were mock-treated (Fig. 4A). WR 7-61-infected mice exhibited increased disease scores at 5 and 6 dpi, with peak average scores of ~1.8 and ~1.3 at 8 and 10 dpi , respectively (Fig. 4B). No alteration in body temperature was observed in the low-dose group. However, in the higher-dose group, mice exhibited a decrease in body temperature between 8 and 9 dpi, with a peak average decline of ~−8 °F (Fig. 4C). Body weight loss was observed in both dose groups, with peak average weight loss at 8 dpi of ~−20% and ~−10% in high- and low-dose groups, respectively (Fig. 4D). Fifty percent of mice infected with 6.0 log_10_ PFU met euthanasia criteria by 9 dpi, whereas all mice infected with 4.0 log_10_ PFU survived the 14-day study (Fig. 4E).

In contrast to infection with WR 7-61, minimal disease was observed in mice infected with MA-2022. Disease scores increased between 1 and 11 dpi, with a peak average score of ~0.8 (Fig. 4B). Minimal or no alterations were observed in body temperature and weight, and all mice survived the 14-day study (Fig. 4C through E).

### Virulence difference between MPXV clade IIa (WR 7-61, US-2003) and IIb (MA-2022) isolates in C57BL/6 *Ifnar*^−/−^ mice via the intranasal route

**Fig 5:**
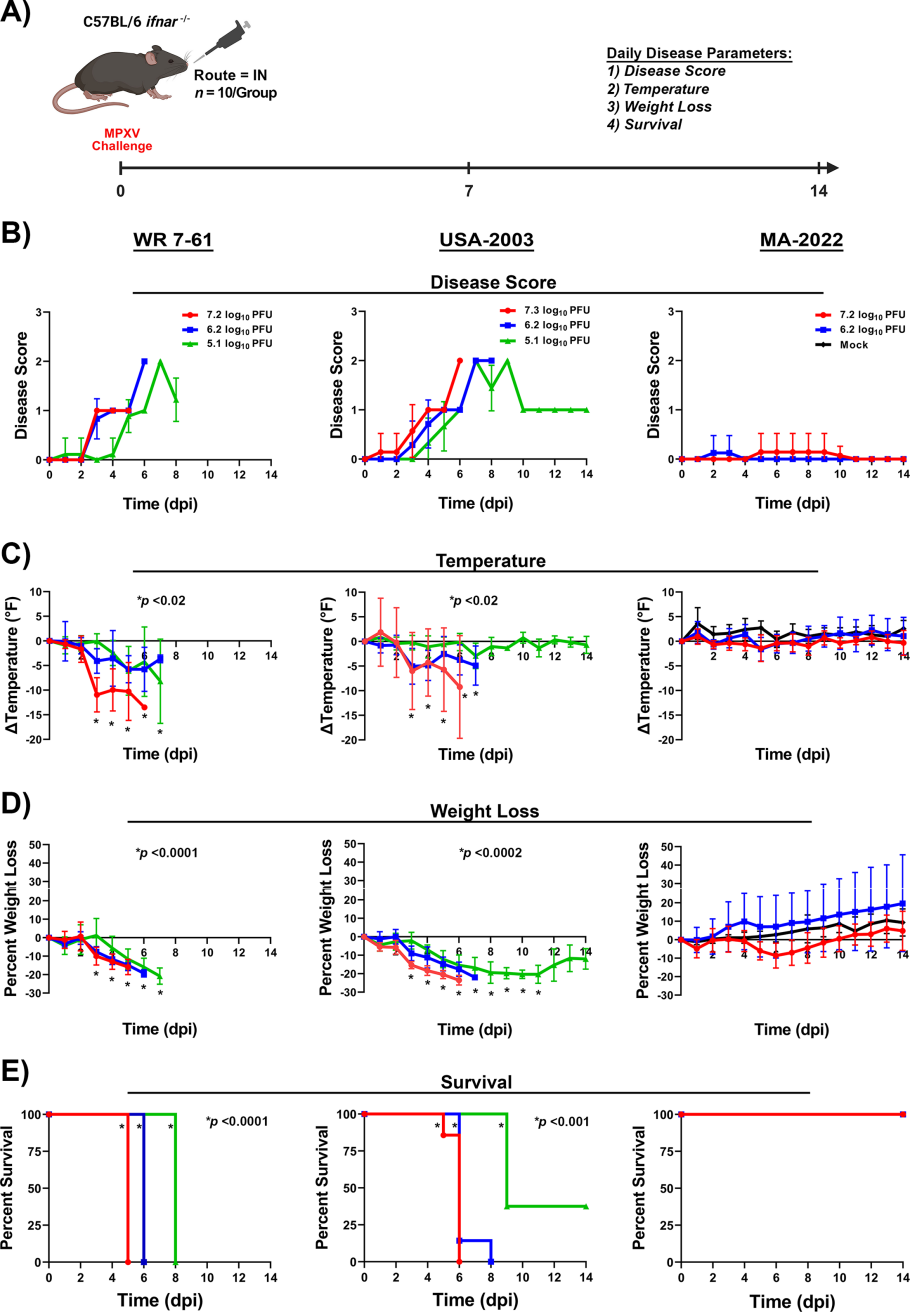
Infection of C57BL/6 *Ifnar^−/^*^−^ mice with MPXV clade IIa (WR 7-61 and US-2003) and IIb (MA-2022) isolates via the intranasal (IN) route. Mice were infected with WR 7-61 and US-2003 at 7.0, 6.0, or 5.0 log_10_ PFU per mouse and with MA-2022 at 7.0 or 6.0 log_10_ PFU per mouse (**A**). Following infection, mice were monitored for disease score (**B**), body temperature (**C**), weight loss (**D**), and survival (**E**). The target dose for each virus was verified by plaque assay, and values are shown (**A**). *P-values comparing MPXV isolates with the mock group are provided in each graph.

To investigate the susceptibility of C57BL/6 *Ifnar*^−/−^ to MPXV infection, cohorts of 10 mice per group were infected via the intranasal route at 7.0, 6.0, or 5.0 log_10_ PFU/mouse (WR 7-61 and US-2003), 7.0 or 6.0 log_10_ PFU/mouse (MA-2022), or were mock-treated (Fig. 5A). As in previous studies, mice were monitored daily following infection for disease score, temperature, weight loss, and survival for 14 days. Disease scores rapidly increased within 3 dpi in mice infected with WR 7-61 and US-2003 at 7.0 and 6.0 log_10_ PFU/mouse dose groups, and, by 4 dpi, all mice exhibited increased disease scores (Fig. 5B). The peak average disease scores were ~1 to ~2 between 5 and 9 dpi in all dose groups. Body temperature decreased rapidly within 2 dpi in all groups infected with WR 7-61 and US-2003, except US-2003 at 5.0 log_10_ PFU (Fig. 5C). The rate of temperature decline was dose dependent, with peak average decline of ~−14°F and ~−10°F in mice infected with WR 7-61 and US-2003 at 7.0 log_10_ PFU/mouse dose, respectively. In all other groups, peak average reduction ranged from ~−3°F to ~−8°F.

Mice infected with WR 7-61 and US-2003 isolates exhibited weight loss within 1 to 3 dpi (Fig. 5D). At 4 dpi, weight loss accelerated in all groups, with peak average weight loss of ~−20% to ~−24%. Only 40% of mice infected with US-2003 at 5.0 Log_10_ PFU/mouse regained weight; however, the weight gain plateaued by the end of the study. All mice infected with WR 7-61 at 7.0, 6.0, or 5.0 log_10_ PFU/mouse dose groups met euthanasia criteria by 5, 6, and 8 dpi, respectively (Fig. 5E). Similarly, all mice infected with US-2003 at 7.0 or 6.0 log_10_ PFU/mouse met euthanasia criteria at 5 to 6 and 6 to 8 dpi, respectively. Partial lethality was observed at 5.0 log_10_ PFU/mouse group, with 60% of the mice meeting euthanasia criteria by 9 dpi.

In contrast to WR 7-61 and US-2003 isolates, infection with MA-2022 was unable to produce severe disease in mice at any dose. Minimal or no changes were observed in disease score and body temperature (Fig. 5B and C). Weight loss was only observed in the 7.0 log_10_ PFU/mouse group between 5 to 9 dpi, with peak average weight loss of ~−9% (Fig. 5D). Mice in the lower dose group did not lose weight throughout the 14-day study. All mice regardless of dose survived the infection with MA-2022 (Fig. 5E).

### Virulence difference between MPXV IIa (WR 7-61, US-2003) and IIb (MA-2022) isolates in C57BL/6 *Ifngr*^−/−^ mice via the intranasal route

**Fig 6:**
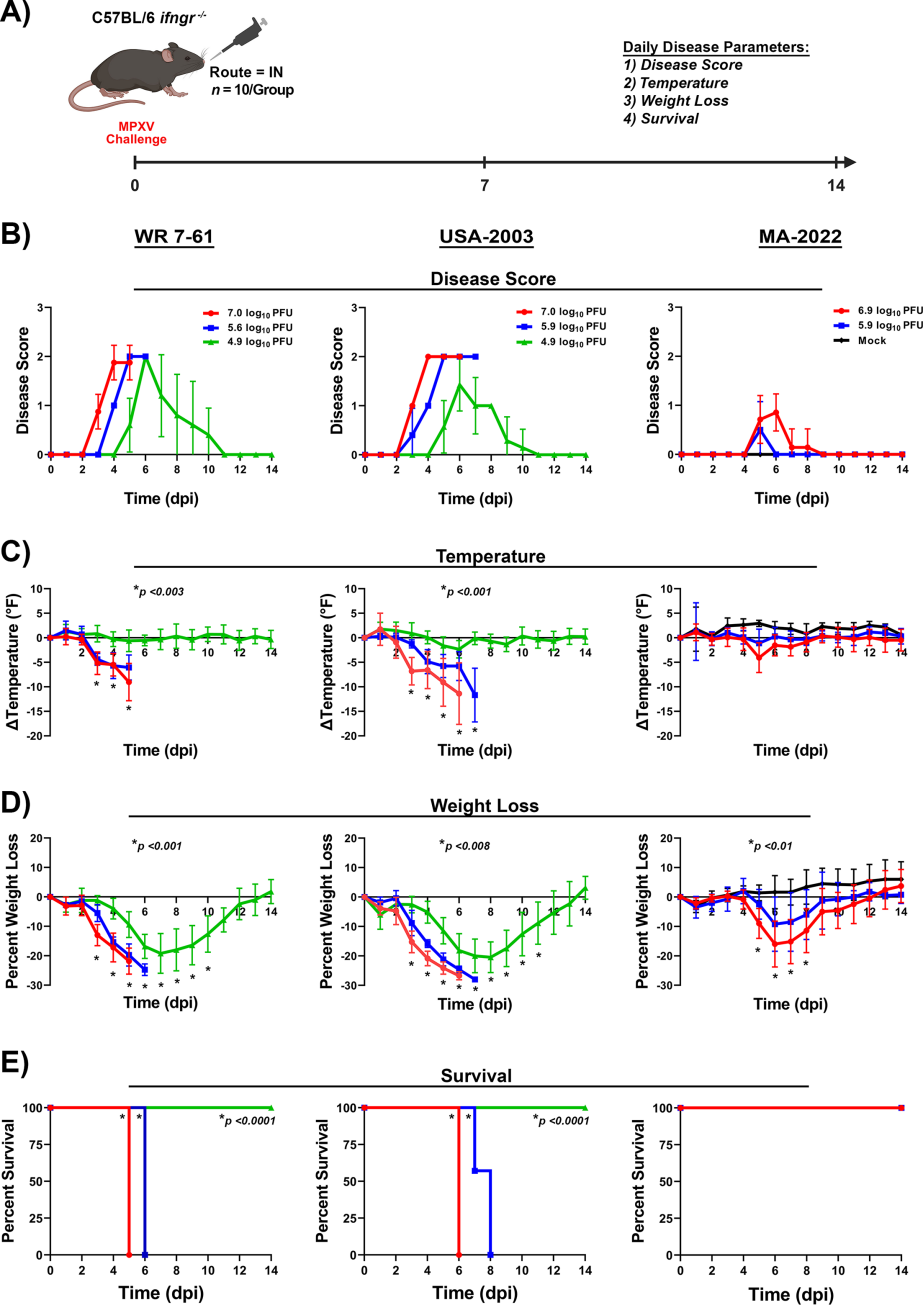
Infection of C57BL/6 *Ifngr^−/−^* mice with MPXV clade IIa (WR 7-61 and US-2003) and IIb (MA-2022) isolates via the intranasal (IN) route. Mice were infected with WR 7-61 and US-2003 at 7.0, 6.0, or 5.0 log_10_ PFU per mouse and with MA-2022 at 7.0 or 6.0 log_10_ PFU per mouse (**A**). Following infection, mice were monitored for disease score (**B**), body temperature (**C**), weight loss (**D**), and survival (**E**). The target dose for each virus was verified by plaque assay, and values are shown (**A**). *P-values comparing MPXV isolates with the mock group are provided in each graph.

All three MPXV isolates were next investigated in the C57BL/6 *Ifngr*^−/−^ background with a similar study design as previous studies (Fig. 6A). Mice infected with the two highest doses of WR 7-61 and US-2003 exhibited a rapid increase in disease score starting at 3 to 4 dpi with peak average score of ~2 (Fig. 6B). The lowest dose groups displayed a 24-h delay in onset of disease signs at 5 dpi. The peak average disease scores were ~1.4 and ~2.0 at 6 dpi and returned to baseline by 11 dpi. Mice infected with the two highest dose groups exhibited a decrease in body temperature starting at 3 dpi (Fig. 6C). Temperature declined peaked between 5 to 7 dpi with peak average decline of ~−9°F to ~−12°F. In contrast, minimal or no decrease in body temperature was observed in mice infected with either isolate at the lowest dose.

All mice infected with WR 7-61 or US-2003 exhibited weight loss, regardless of dose group (Fig. 6D). Mice exhibited weight loss starting at 1 dpi, with peak average weight loss of ~−19% to ~−28% at 5 to 8 dpi. Mice in the lowest dose groups regained weight between 8 to 9 dpi and were at or above the initial weight by 14 dpi. All mice in two highest dose groups met euthanasia criteria between 5 and 8 dpi (Fig. 6E). In contrast, all mice infected with either isolate at the lowest dose survived the 14-day study.

Mice infected with the MA-2022 isolate exhibited mild disease in both dose groups. Disease scores increased on 5 dpi, with peak average scores of ~0.9 and ~0.5, and returned to baseline by 9 (high dose) and 6 dpi (low dose), respectively (Fig. 6B). Body temperature declined at the high dose between 5 to 8 dpi, with peak average decline of ~−4°F, and by 9 dpi, all mice returned to baseline (Fig. 6C). All mice in both dose groups exhibited weight loss in a dose-dependent manner beginning at 5 dpi, with peak average weight loss ranging from ~−16% to ~−9% at 6 dpi (Fig. 6D). Mice began to regain weight by 7 dpi and returned to baseline by 12 (high dose) and 10 dpi (low dose). All mice infected with the MA-2022 isolate survived the 14-day study (Fig. 6E).

### Virulence difference between MPXV clade IIa (WR 7-61, US-2003) and IIb (MA-2022) isolates in C57BL/6 *Ifnar*^−/−^/*Ifngr*^−/−^ mice via the intranasal route

**Fig 7:**
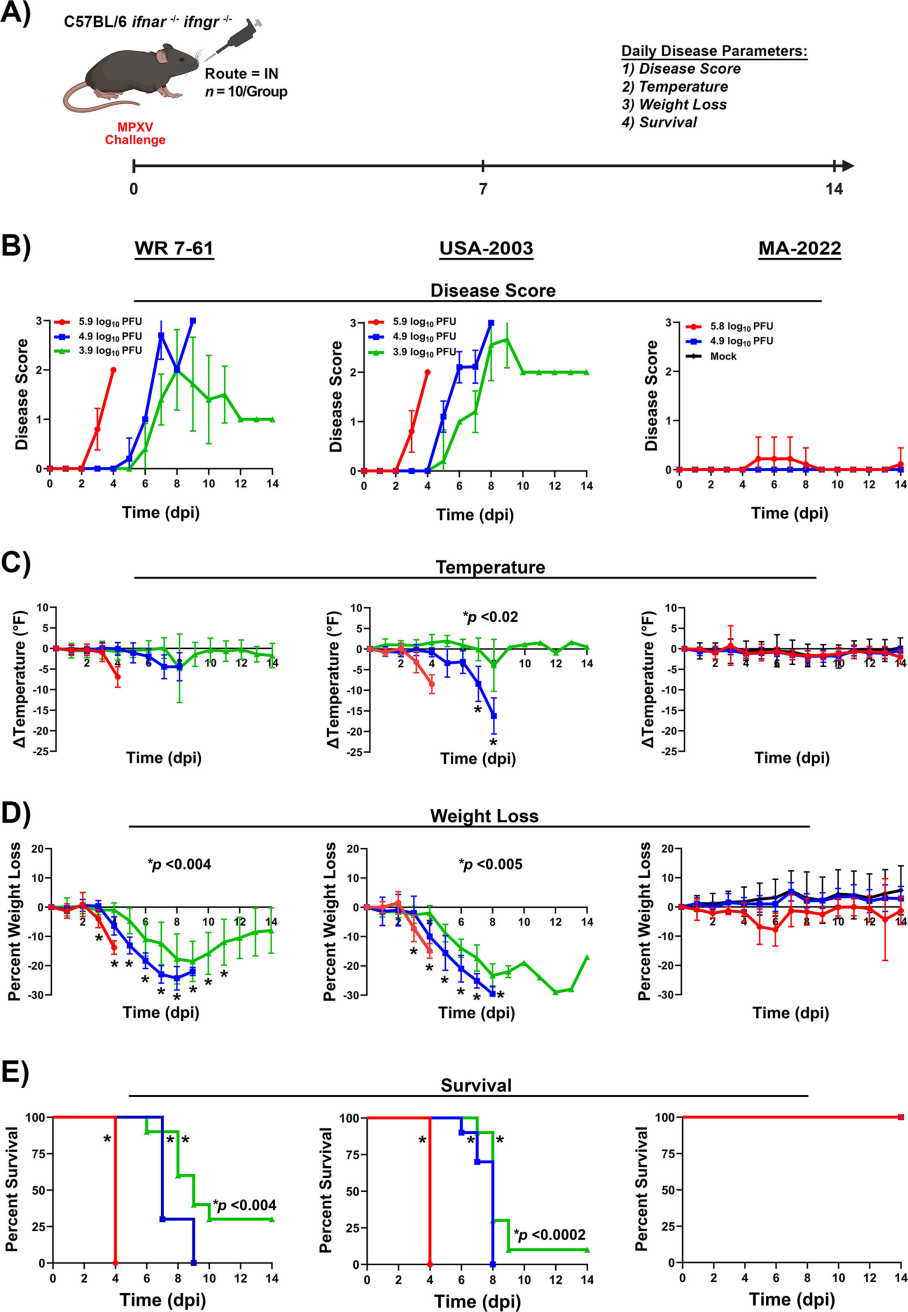
Infection of C57BL/6 *Ifnar^−/−^/ifngr^−/−^* mice with MPXV clade IIa (WR 7-61 and US-2003) and IIb (MA-2022) isolates via the intranasal (IN) route. Mice were infected with WR 7-61 and US-2003 at 6.0, 5.0, or 4.0 log_10_ PFU per mouse and with MA-2022 at 6.0 or 5.0 log_10_ PFU per mouse (**A**). Following infection, mice were monitored for disease score (**B**), body temperature (**C**), weight loss (**D**), and survival (**E**). The target dose for each virus was verified by plaque assay, and values are shown (**A**). *P-values comparing MPXV isolates with the mock group are provided in each graph.

The study design in C57BL/6 *Ifnar*^−/−^/*Ifngr*^−/−^ mice was similar to that in previous studies. Mice infected with WR 7-61 and US-2003 isolates received three doses (6.0, 5.0, or 4.0 log_10_ PFU/mouse), whereas MA-2022-infected mice received two doses (6.0 or 5.0 log_10_ PFU/mouse) (Fig. 7A). WR 7-61- and US-2003-infected mice at 6.0 log_10_ PFU/mouse exhibited severe disease, with rapid alterations in disease score, body temperature, and weight loss between 3 and 4 dpi (Fig. 7B through D). Disease score increased at 3 dpi, with a peak average score of ~2 at 4 dpi. The peak decline in average body temperature ranged from ~−7°F to ~−9°F. Mice exhibited weight loss ranging from ~−14% to ~−15%, and all mice met euthanasia criteria by 4 dpi.

Mice infected with WR 7-61 and US-2003 at lower doses exhibited a delayed disease kinetics by 1 to 2 days. Disease scores increased between 5 and 6 dpi, with peak scores of ~3 in the intermediate and ~2 to ~2.7 in the lowest-dose groups (Fig. 7B). Body temperature began to decline between 6 and 8 dpi (Fig. 7C). In the intermediate-dose group, the temperature decline was more pronounced in US-2003-infected mice (~−16°F) than in WR 7-61-infected mice (~−5°F). The temperature decreased in the lowest-dose groups at 8 dpi with average decline of ~−5°F; however, the temperature returned to baseline level within 24 h. Weight loss in intermediate- and lowest-dose groups exhibited similar kinetics, with the on-set of weight loss between 4 and 5 dpi (Fig. 7D). However, the magnitude of weight loss was greater in US-2003-infected mice than WR 7-61-infected mice. At intermediate- and lowest-US-2003-dose groups, peak average weight loss was ~−30%. In contrast, the intermediate and lowest WR 7-61 dose groups had weight loss of ~−25% and ~−19%, respectively. All mice infected with either isolate at the intermediate dose met euthanasia criteria between 6 and 9 dpi (Fig. 7E). At the lowest dose, 70% of WR 7-61-infected mice and 90% of US-2003-infected mice met euthanasia criteria between 6 and 10 dpi.

Similar to C57BL/6 *Ifnar*^−/−^ and *Ifngr*^−/−^ mouse studies, the MA-2022 isolate did not produce severe disease in C57BL/6 *Ifnar*^−/−^/*Ifngr*^−/−^ mice. Minimal or no changes were observed in disease score or body temperature (Fig. 7B and C). In the high-dose group, modest weight loss of ~−8% was observed between 4 and 5 dpi (Fig. 7D). The low-dose group exhibited no weight loss and was indistinguishable from the mock infected group. All MA-2022-infected mice survived the 14-day study (Fig. 7E).

**Fig 8:**
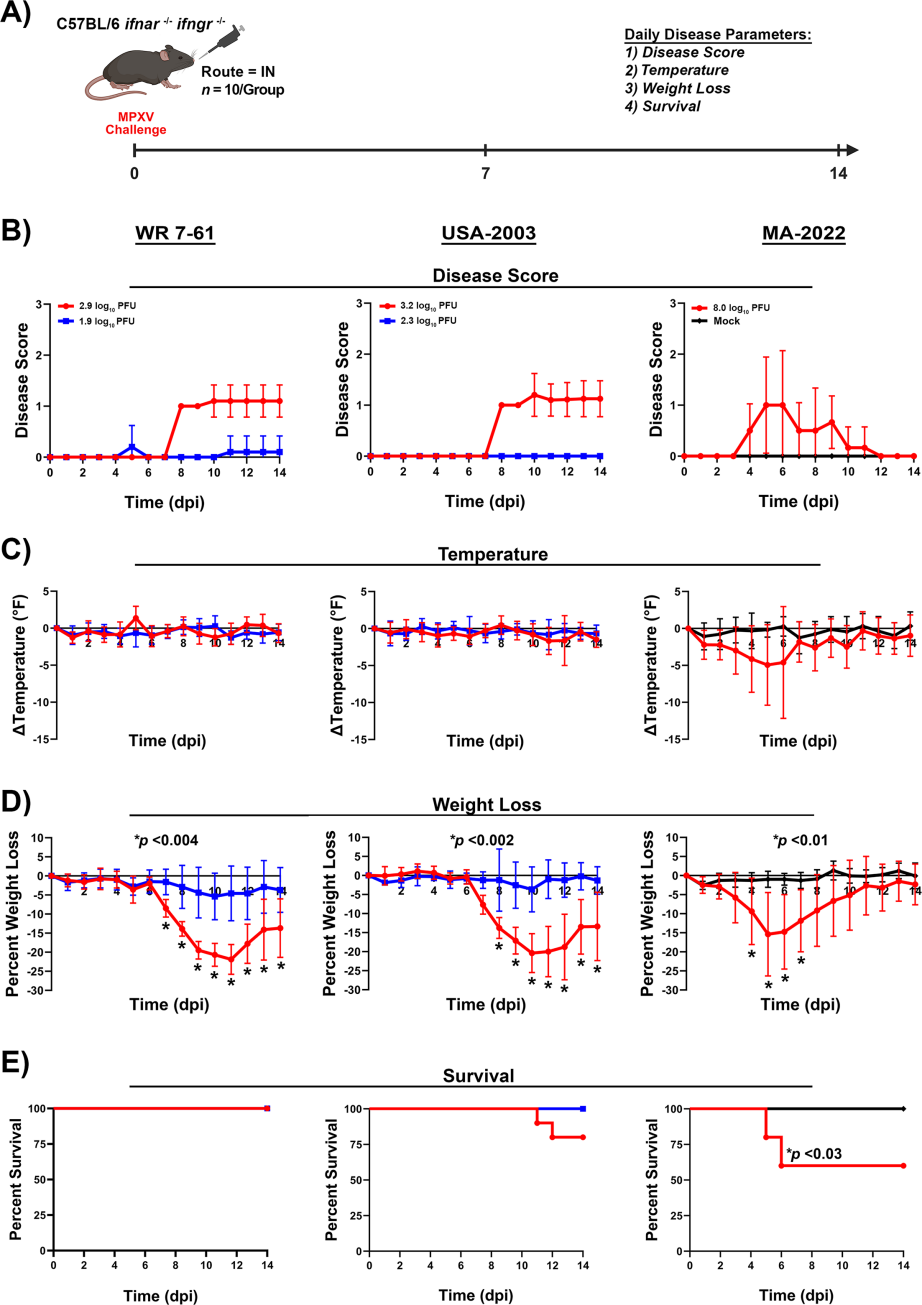
Infection of C57BL/6 *Ifnar^-/-^/Ifngr^-/-^* mice with MPXV clade IIa (WR 7-61 and US-2003) and IIb (MA-2022) isolates via the intranasal (IN) route. Mice were infected with WR 7-61 and US-2003 at 3.0 or 2.0 log_10_ PFU per mouse and with MA-2022 at 8.0 log_10_ PFU per mouse (**A**). Following infection, mice were monitored for disease score (**B**), body temperature (**C**), weight loss (**D**), and survival (**E**). The target dose for each virus was verified by plaque assay, and values are shown (**A**). *P-values comparing MPXV isolates with the mock group are provided in each graph.

A follow-up infection study in C57BL/6 *Ifnar*^−/−^/*Ifngr*^−/−^ mice was conducted at 3.0 or 2.0 log_10_ PFU (WR 7-61 and US-2003) and 8.0 log_10_ PFU (MA-2022) (Fig. 8A).

Mice infected with WR 7-61 and US-2003 at 3.0 log_10_ PFU exhibited disease scores from 7 to 14 dpi with peak scores of ~1.2 (Fig. 8B). Only minimal alterations were observed in body temperature (Fig. 8C). Body weight declined at 7 dpi, with peak weight loss of ~−21% between 10 and 11 dpi and did not return to baseline at 14 dpi (Fig. 8D). All mice infected with WR 7-61 survived the infection, whereas 20% of mice infected with US-2003 met the euthanasia criteria. Infection of mice with WR7-61 and US-2003 at a dose of 2.0 log_10_ PFU produced minimal disease. Minimal or no alterations in disease scores or body temperature were observed (Fig. 8B and C). A modest decrease in body weight was observed between 9 and 14 dpi, with peak decline of ~−5% (Fig. 8D). All mice survived the 14-day study (Fig. 8E).

Mice infected with MA-2022 at 8.0 log_10_ PFU developed severe disease. Disease scores began to increase at 4 dpi and peaked at between 5 and 6 dpi, with average score of ~1 (Fig. 8B). Body temperature and weight started to decline at 1 and 3 dpi, with peak decline of ~−5°F and ~−15% at 5 dpi (Fig. 8C and D). Forty percent of the mice met euthanasia criteria between 5 and 6 dpi (Fig. 8E).

### Susceptibility of C57BL/6 *Ifnar*^−/−^ mice to MPXV clade IIa (WR 7-61) and IIb (MA-2022) isolates via the intravaginal and intrarectal routes

**Fig 9:**
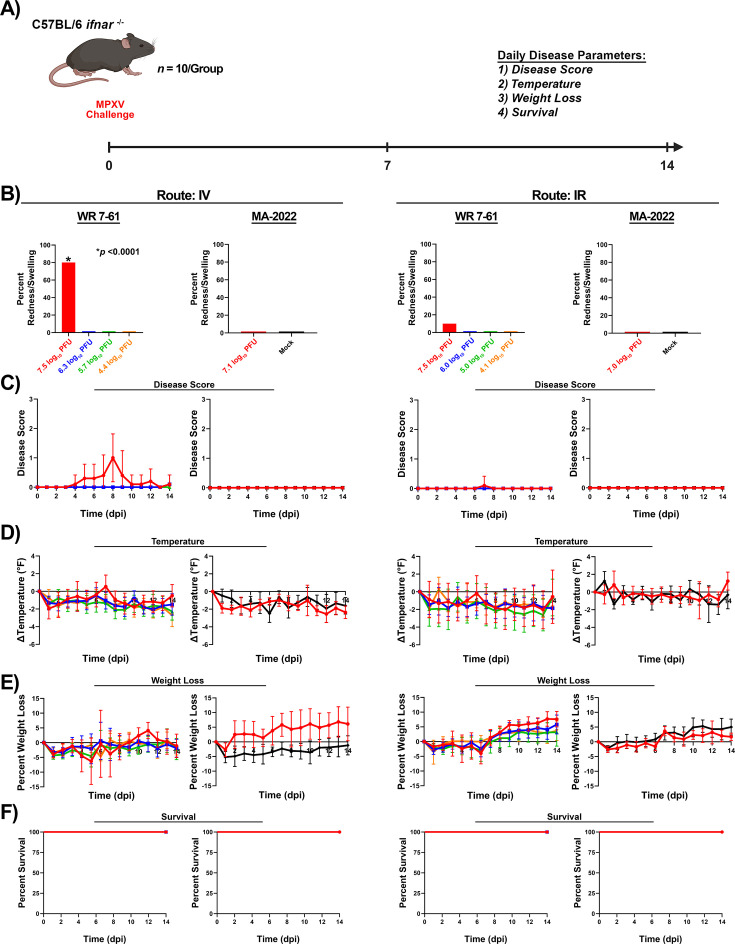
Infection of C57BL *ifnar^−/^*^−^ mice with MPXV clade IIa (WR 7-61) or IIb (MA-2022) isolates via the intravaginal (IV) and intrarectal (IR) routes. Mice were infected with WR 7-61 at 7.0, 6.0, 5.0, or 4.0 log_10_ PFU per mouse and with MA-2022 at 7.0 log_10_ PFU per mouse (**A**). Following infection, mice were monitored for swelling and redness at challenge site (**B**), disease score (**C**), body temperature (**D**), weight loss (**E**), and survival (**F**). The target dose for each virus was verified by plaque assay, and values are shown (**B**). *P-values comparing MPXV isolates with the mock group are provided in each graph.

C57BL/6 *Ifnar*^−/−^ mice were infected via the intravaginal (IV) or intrarectal (IR) route with WR 7-61 at 7.0, 6.0, 5.0, or 4.0 log_10_ PFU/mouse, or with MA-2022 at 7.0 log_10_ PFU/mouse, or mock treated (Fig. 9A). Following infection with WR 7-61 at 7.0 log_10_ PFU/mouse via the IV route, mice exhibited redness and/or swelling at the site of challenge starting at 4 dpi. Eighty percent of mice exhibited vaginal redness and/or swelling with a peak average disease score of ~1 at 8 dpi (Fig. 9B and C). The redness and swelling were largely resolved by 14 dpi. In contrast, no overt disease was observed in the lower dose groups. Body temperature varied by ~−1°F to ~−2°F throughout the post-challenge period in all mice infected with WR 7-61, a pattern similar to that of the mock treated group (Fig. 9D). Mice exhibited weight loss in a dose-dependent manner beginning at 2 dpi, with peak weight loss values of ~−3% to ~-6% between 1 to 5 dpi (Fig. 9E). All mice, regardless of dose, survived the 14-day study (Fig. 9F).

Infection of mice with WR 7-61 via the IR route produced minimal or no redness/swelling at the site of infection, changes in disease score, or alterations in body temperature (Fig. 9B through D). However, mice did exhibit weight loss between 1 and 6 dpi with peak values ranging from ~−2% to ~−4% (Fig. 9E). Similar to the IV challenge, all mice survived the 14-day study (Fig. 9F).

Infection with MA-2022 via the IV or IR routes did not exhibit any overt disease or alteration in body temperature (Fig. 9B through D). Mice exhibited minimal weight loss and survived the 14-day study (Fig. 9E through F).

### Susceptibility of C57BL/6 *Ifnar*^−/−^/*Ifngr*^−/−^ mice to MPXV clade IIa (WR 7-61) and IIb (MA-2022) isolates via the intravaginal and intrarectal routes

**Fig 10:**
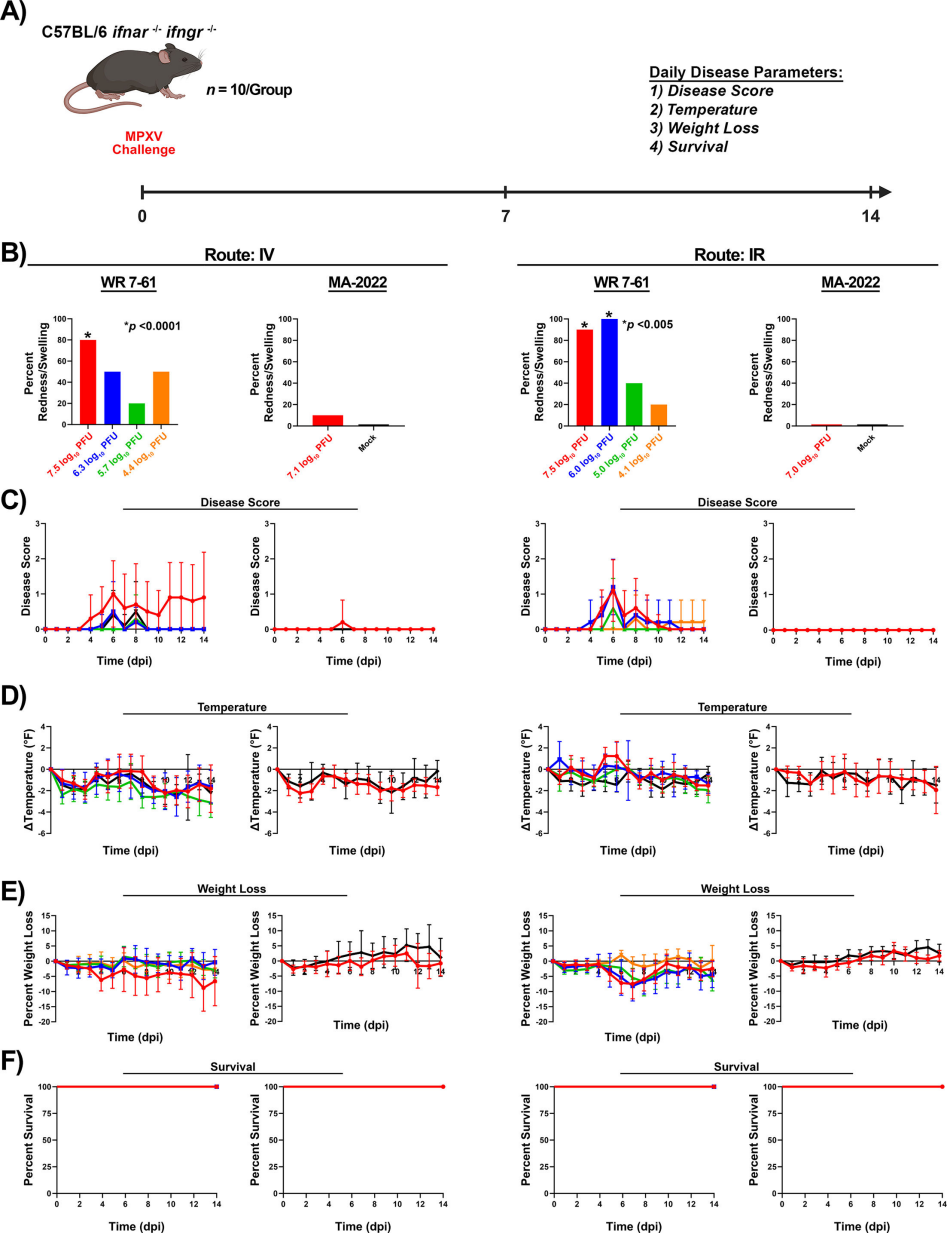
Infection of C57BL/6 *Ifnar^−/−^/Ifngr^−/−^* mice with MPXV clade IIa (WR 7-61) or IIb (MA-2022) isolates via the intravaginal (IV) and intrarectal (IR) routes. Mice were infected with WR 7-61 at 7.0, 6.0, 5.0, or 4.0 log_10_ PFU per mouse and with MA-2022 at 7.0 log_10_ PFU per mouse (**A**). Following infection, mice were monitored for swelling and redness at challenge site (**B**), disease score (**C**), body temperature (**D**), weight loss (**E**), and survival (**F**). The target dose for each virus was verified by plaque assay, and values are shown (**B**). *P-values comparing MPXV isolates with the mock group are provided in each graph.

The susceptibility via the IV and IR routes was investigated in C57BL/6 *Ifnar*^−/−^/*Ifngr*^−/−^ mice with WR 7-61 at 7.0, 6.0, 5.0, or 4.0 log_10_ PFU/mouse, or MA-2022 at 7.0 log_10_ PFU/mouse, or mock treated (Fig. 10A). Twenty to eighty percent of mice infected with WR 7-61 via the IV route exhibited redness and/or swelling at the site of challenge (Fig. 10B). Mice infected with the highest dose exhibited disease scores throughout the 14-day challenge study, starting at 4 dpi with an average peak disease score of ~1 (Fig. 10C). Infection at lower doses produced disease scores at 6 and 8 dpi, with average peak score of ~0.5. Body temperature in all dose groups displayed minimal alterations and exhibited a similar pattern to that of the mock group (Fig. 10D). Body weight loss was observed in the highest dose group, with peak average weight loss of ~−9% at 13 dpi (Fig. 10E). Minimal weight loss was detected in all other dose groups. All mice, regardless of dose, survived the 14-day study (Fig. 10F).

Infection with the WR 7-61 isolate via the IR route produced redness and/or swelling in 20% to 100% of the mice in a dose-dependent manner (Fig. 10B). Mice exhibited disease scores between 4 and 14 dpi, with peak scores ranging from ~0.3 to ~1.2 (Fig. 10C). The pattern of variation in body temperature was similar to that of mock-infected mice across all dose groups (Fig. 10D). Body weight loss displayed a similar pattern in three of the highest dose groups (Fig. 10E). The mice exhibited weight loss starting at 5 dpi, with peak weight of ~−7% to ~-8% between 7 and 8 dpi. Minimal weight loss was observed in the lowest-dose group. Similar to the IV challenge study, all mice survived the 14-day study (Fig. 10F).

In contrast to infection with WR 7-61, mice infected with MA-2022 via the IV or IR routes exhibited minimal or no disease, alteration in body temperature, minimal weight loss, and all mice survived the 14-day study (Fig. 10B through F).

## DISCUSSION

Despite the threats that mpox poses to human health, few murine models are available for investigating the pathogenesis of emerging MPXV clades. Four mouse strains including suckling white mice, SCID-BALB/c, C57BL/6 *stat1^−/^*^−^, and CAST/EiJ, have been shown to be susceptible to MPXV infection and exhibit lethal disease (6, 45–48). Of these models, the immune system of suckling mice and SCID-BALB/c is either not fully functional at the time of infection or is lacking. In contrast, both C57BL/6 *stat1^−/−^* and CAST/EiJ have deficiencies in the interferon response, which enables susceptibility to MPXV infection and disease. However, despite these deficiencies, both models can generate immune responses, and have been utilized in viral pathogenesis and countermeasure development studies (48–50).

Of the two murine models, only the CAST/EiJ has been used in previous comparative MPXV virulence studies (6, 45). The clade Ia Zaire-79-CB2 isolate (clonal or uncloned) was shown to be 2- to 10-fold more virulent than the clade IIa US-2003 isolate via the intranasal route (6, 45). The clade IIb isolate MA-2022 was unable to cause severe disease via either intranasal or intraperitoneal routes and was 100-fold less virulent than the US-2003 isolate (45). These studies demonstrated significant differences in the virulence of MPXV clades, with clade Ia exhibiting 1,000-fold greater virulence than clade IIb.

The present study investigated differences in the virulence of clade IIa (WR 7-61) and IIb (MA-2022) isolates in CAST/EiJ mice via the intranasal route. WR 7-61 isolate caused severe disease including lethality at 7.0, 5.0, and 4.0 log_10_ PFU in 7- to 8-week-old mice and at 6.0 log_10_ PFU in 4- to 5-month-old mice. In contrast, the MA-2022 isolate was unable to cause severe disease at 7.0 log_10_ PFU, regardless of age. These data demonstrate that the MA-2022 isolate is at least 1,000-fold less virulent than the WR 7-61 isolate. In addition, these data corroborate previous clade II results and extend the findings in the CAST/EiJ model (39). Lastly, the susceptibility of 4- to 5-month-old CAST/EiJ mice to clade IIa infection highlights the utility of this model for evaluating long-term vaccine durability and efficacy.

Although the CAST/EiJ mouse model is susceptible to MPXV infection, it has several important limitations. First, commercial vendors have limited quantities, and large orders cannot be accommodated, particularly during outbreaks or in projects with short timelines. Second, this mouse strain exhibits low pregnancy rates, small litter sizes, and high rates of maternal cannibalism. As a result of these traits, our attempt to establish an internal colony over approximately 1.5 years yielded poor results. Third, mice are highly fractious, and additional ABSL-2 and -3 personnel and safety procedures are required for breeding and/or experimental studies. For these reasons, only limited pilot studies with five to six animals per group were conducted, consequently warranting the investigation of additional mouse models.

Interferon alpha (IFN-α) and gamma (IFN-γ) play critical roles in the host response against poxvirus infection, and the disruption of either pathway dramatically increases the susceptibility of mice. Previous studies explored C57BL/6 *stat1^−/−^* mice that lack the STAT1 protein, which is essential for transmitting signals from type I and II interferon receptors to the nucleus, resulting in a diminished antiviral immune response. Similarly, CAST/EiJ mice exhibit a deficient IFN-γ response, thus impairing an effective immune response against MPXV. In particular, a previous study investigated the susceptibility of CAST/EiJ mice to MPXV and found that the absence or reduced production of IFN-γ severely impaired the ability to control viral replication (49). As a result, MPXV spread unchecked, producing severe systemic infection leading to lethality. The study also highlighted that CAST/EiJ mice failed to upregulate critical immune pathways dependent on IFN-γ, which is central to activating macrophages, promoting antigen presentation, and inducing antiviral state in infected cells. In a follow-up study delving further into the underlying mechanisms, the research demonstrated that these mice not only have an impaired IFN-γ response but also show insufficient innate immunity characterized by low levels of Natural Killer (NK) cells following infection (50). NK cells are critical components of the early innate immune response responsible for recognizing and eliminating virus-infected cells prior to the activation of the adaptive immune system. The mitigation of this defect via administration of NK cells or by the pre-stimulation of the interferon pathways led to improved outcomes. Pre-activation of interferon signaling reduced virus replication and disease severity while increasing survival rates. These studies highlight the importance of IFN in the host antiviral response against MPXV infection in mice.

Due to the critical role of IFN-α and IFN-γ, we investigated the susceptibility of three murine models, C57BL/6 *Ifnar*^−/−^, *Ifngr*^−/−^, and *Ifnar*^−/−^/*Ifngr*^−/−^, which lack functional type I and/or II interferon receptors. In addition to IFN deficiencies, these mouse strains are readily available through commercial vendors and more amenable to the establishment of breeding colonies than CAST/EiJ mice. All three mouse strains were susceptible to clade IIa infection. The WR 7-61 and US-2003 isolates caused severe disease at doses ranging from 5.0 to 7.0 log_10_ PFU in C57BL/6 *Ifnar*^−/−^ and *Ifngr*^−/−^ mice. Not surprisingly, both isolates caused severe disease at 3.0 to 6.0 log_10_ PFU in the most immunocompromised murine model, C57BL/6 *Ifnar*^−/−^/*Ifngr*^−/−^ mice. In contrast to the clade IIa isolates, the clade IIb MA-2022 isolate did not cause severe disease at doses ranging from 5.0 to 7.0 log_10_ PFU. The MA-2022 isolate caused severe disease only at 8.0 log_10_ PFU dose in the C57BL/6 *Ifnar*^−/−^/*Ifngr*^−/−^ mice. These data demonstrate that the C57BL/6 *Ifnar*^−/−^, *Ifngr*^−/−^, and *Ifnar*^−/−^/*Ifngr*^−/−^ mice are susceptible to MPXV clade II infection, and the clade IIb MA-2022 isolate from the 2022 outbreak is ~100 to 100,000-fold less virulent than either clade IIa isolates (WR 7-61 or US-2003).

This study also investigated the susceptibility of C57BL/6 *Ifnar*^−/−^ and C57BL/6 *Ifnar*^−/−^/*Ifngr*^−/−^ mice to MPXV infection via the intravaginal and intrarectal routes. Infection with either WR 7-61 or MA-2022 was unable to produce severe disease in both mouse strains. However, mice infected with WR 7-61 exhibited weight loss and redness/swelling at the sites of infection, particularly via the intrarectal route. The redness/swelling was more pronounced in C57BL/6 *Ifnar*^−/−^/*Ifngr*^−/−^ mice at the infection site via both routes. In contrast, mice infected with MA-2022 did not exhibit any overt disease. These data further highlight the differences in virulence between clade IIa and IIb isolates.

IFN-α and/or IFN-γ receptor knockout mice have been previously investigated and reported to be resistant to MPXV clade Ia infection (48). The data from the present study do not support the conclusions of the prior study. Several potential explanations may account for these conflicting results. First, the dose of MPXV clade Ia utilized in the previous study may be too low (~3.0 log_10_ PFU) to investigate susceptibility. Second, the receptor knockout approaches may not have completely eliminated interferon receptor function, and the residual activity enabled resistance to infection. Third, the knockout mouse strains may have exhibited elevated basal levels of cytokines, such as TNF-α, IL-1, and MIP-1α, which could have limited orthopoxvirus replication (62). However, these hypotheses require further investigation to reconcile the inconsistencies between the murine models.

To investigate the genetic basis of virulence differences among MPXV clades Ia, IIa, and IIb, we performed a comprehensive comparative genomic analysis (Table 1). Orthopoxviruses, including cowpox, horsepox, monkeypox, vaccinia, and variola comprise large genomes ranging from ~167 to 225 kbp dsDNA genomes encoding ~170 to 214 genes (1, 63). The central ~100 kbp core encodes genes responsible for virus replication and is consequently highly conserved. In contrast, the ~30 to 40 kbp on either left or right arm of the genome is more divergent and encodes host range restriction and host immune antagonism genes. The more virulent clade Ia encodes multiple host antagonism genes (D14L and B14R) that are absent or truncated in both clades IIa and IIb. D14L (C3L ortholog) encodes a complement binding protein that is a potent inhibitor of the complement-mediated antiviral response (27, 64–68). In addition, the B14R gene encodes an IL-1 receptor homolog that inhibits host inflammatory response and is only present in clade Ia (69). Clade IIa encodes two additional virulence factors, N3R (OPG0165) and N1R (OPG005), which are absent in the less virulent clade IIb. N3R encodes a secreted MHC Class I homolog that binds to and blocks the function of the NKG2D receptor, thereby reducing the activation of Natural Killer and T-cells (70). N1R encodes a protein with homology to the anti-apoptotic BCL-2 domain (71, 72). The presence or absence of these genes may contribute to the virulence differences between clades Ia, IIa, and IIb. In addition to these genes, comparative analyses have identified amino acid substitutions, insertions, and deletions, the significance of which is difficult to assess without comprehensive studies. Further studies are required to fully elucidate critical gene/s that underlie the virulence difference between MPXV clades.

To further characterize the viral stocks used in these studies, all three sucrose purified stocks were full-length sequenced via next-generation sequencing (NGS). The sequencing analysis identified single-nucleotide polymorphisms (SNPs) in each isolate relative to its corresponding reference genome sequence. WR 7-61 and US-2003 isolates exhibited substantially more SNPs than the MA-2022 isolate (Tables S1a; S1b). This likely reflects differences in virus amplification history among clade II isolates. WR 7-61 is one of the oldest MPXV clade II isolates, originally obtained in 1961 at the Walter Reed Army Institute of Research (WRAIR) (18). The virus was isolated from a skin lesion on a female cynomolgus macaque that developed mpox disease 45 days after whole-body irradiation. The isolate was initially propagated in monkey kidney cells, plaque-purified three times in rabbit kidney cells, and further passaged in rabbit kidney and Vero cells (18, 26). The US-2003 isolate was obtained during the 2003 mpox outbreak from Wisconsin, USA (73). The virus was isolated from a skin lesion of an infected human and subsequently passaged in BSC-40 and MA-104 clone 1 cells (25). In contrast, the MA-2022 isolate was derived from a lesion swab collected from an infected individual during the 2022 outbreak and underwent comparatively limited *in vitro* passaging. In addition, the identification of SNPs may be due to different sequencing technologies used to generate the reference sequences. The clade IIa isolates were originally sequenced in 2005 via Sanger sequencing; in contrast, the clade IIb isolate was sequenced using NGS technology during the 2022 outbreak (25, 26, 65). Consequently, the NGS analysis identified additional SNPs in WR 7-61 and US-2003 isolates than their Sanger-derived reference sequences. Despite the higher number of SNPs, the majority of variants were located within open reading frames (ORFs) with limited or uncharacterized functional annotation. Nonetheless, the contribution of SNPs to the virulence of clade IIa isolates warrants further investigation.

Lastly, there are several important limitations to our study. First, the virulence of clades Ia and Ib was not investigated in immunocompromised murine models. Clade Ia is more virulent than clade II in mice and would presumably exhibit greater virulence in the immunocompromised models. Second, whether immunocompromised models can be utilized in the context of mpox vaccine studies remains to be determined. Further studies are underway to address these limitations.

In summary, we investigated differences in the virulence of clade IIa and IIb isolates in CAST/EiJ, C57BL/6 *Ifnar*^−/−^, *Ifngr*^−/−^, and *Ifnar*^−/−^/*Ifngr*^−/−^ mice. In CAST/EiJ mice, the clade IIb MA-2022 isolate was up to 1,000-fold less virulent than clade IIa WR 7-61 isolate. Moreover, we demonstrate that three additional mouse models (C57BL/6 *Ifnar*^−/−^, *Ifngr*^−/−^, and *Ifnar*^−/−^/*Ifngr*^−/−^) are susceptible to MPXV clade II infection. Lastly, the MA-2022 isolate exhibited ~100 to 100,000-fold lower virulence than clade IIa isolates in C57BL/6 *Ifnar*^−/−^, *Ifngr*^−/−^, and *Ifnar*^−/−^/*Ifngr*^−/−^ mice. Taken together, these data highlight differences in the virulence of clade II isolates and provide three additional murine models to investigate MPXV infection and pathogenesis.

## MATERIALS AND METHODS

### Genetic and phylogenetic analysis

Genetic analysis was performed by downloading MPXV sequences and aligning them in Geneious Prime (version 2025.2). Phylogenetic analysis was performed by downloading 18 nonredundant genome sequences representing main clades of MPXV from GenBank. All sequences were subsequently aligned with MAFFT v7 (FFT-NS-i strategy as iterative refinement method) (74). A maximum likelihood phylogenetic tree was reconstructed for the aligned data set using PhyML under the GTR + GAMMA (4 CAT) substitution model (75).

### Cells

Vero (CRL-1586) and BSC-40 (CRL-2761) cells were obtained from American Tissue Culture Collection (ATCC, Manassas, VA). Cells were cultured in Dulbecco’s Modified Eagle Medium (DMEM) supplemented with 10% fetal bovine serum (FBS) and gentamicin (50 µg/mL) at 37°C and 5% CO_2_.

### Viruses and virus amplification

MPXV clade IIa (WR7-61 and US-2003) and IIb (MA-2022) isolates were obtained from BEI Resources (Manassas, VA). Virus stocks of each isolate were generated on BSC-40 cells. Briefly, cells were seeded overnight in flasks to achieve 70% confluence, growth media was removed, and monolayers were infected at a multiplicity of infection (MOI) of 0.01. Following an hour incubation at 37°C and 5% CO_2_, 20 mL of growth media was added. Monolayers were harvested via cell scraping 72 h post-infection (hpi). Samples were subjected to three cycles of freeze/thaw/sonication and stored at −80°C. For murine studies, virus stocks were sucrose purified. All virus stocks were titrated by plaque assay on BSC-40 cells.

### Virus titration

BSC-40 cells were seeded overnight in six-well plates to achieve 95–100% confluence. Each virus stock was serially diluted 10-fold in 1× DMEM. Monolayers were infected with 100 µL of each dilution and incubated at 37°C and 5% CO_2_ for 1 h. Following incubation, monolayers were overlaid with 2 mL of 0.4% methylcellulose in MEM, GlutaMAX (Gibco) supplemented with 5% FBS and gentamicin (50 µg/mL). Following 72 h at 37°C and 5% CO_2_, plates were fixed in 10% formalin overnight. Plates were stained with 2% crystal violet in 70% ethanol for 5–10 min. The stain was removed with water and dried, and plaques were counted. For plaque phenotype comparison, an identical protocol was performed with Vero and BSC-40 cells, and plaques were imaged at 96 hpi.

### Multi-step growth kinetics

BSC-40 cells were seeded overnight in six-well plates to achieve 50% confluence. Growth media was removed, and triplicate monolayers were infected at an MOI of 0.01 with MPXV isolates. Following an hour incubation at 37°C and 5% CO_2_, monolayers were washed three times with 10 mL PBS, and 2 mL of growth media was added. Monolayers were harvested via cell scraping at 0, 24, 48, 72, and 96 hpi. Samples were subjected to three cycles of freeze/thaw/sonication and stored at −80°C.

### Mouse studies

Breeding pairs of CAST/EiJ, C57BL/6 *Ifnar^−/−^*, *Ifngr^−/−^*, *Ifnar^−/−^*/*Ifngr ^−/−^* mice were purchased from Jackson Laboratories to establish mouse colonies. Fourteen-day studies were performed to investigate virulence differences between the MPXV clade IIa (WR 7-61 and US-2003) and IIb (MA-2022) isolates. For CAST/EiJ studies, 7- to 8-week and 4- to 5-month-old mice were utilized with 5 to 10 mice per group. Mice were infected with WR 7-61 (7.0, 6.0, 5.0, or 4.0 log_10_ PFU) and MA-2022 (7.0 log_10_ PFU) via the intranasal route. Mice were infected with 30 µL (15 µL/nare) of virus or 1× PBS. For C57BL/6 *Ifnar^−/−^*, *Ifngr^−/−^*, *Ifnar^−/−^*/*Ifngr^−/−^* mouse studies, cohorts of 10 (five male and five female) 6- to 8-week-old mice were infected with MPXV isolates via the intranasal route. Mice were infected with 30 µL (15 µL/nare) of virus or 1× PBS. C57BL/6 *Ifnar^−/−^* and *Ifngr^−/−^* mice were infected with three different doses, 7.0, 6.0, or 5.0 log_10_ PFU/mouse, with WR 7-61 and US-2003 isolates. Both strains were also infected with MA-2022 at 7.0 or 6.0 log_10_ PFU/mouse. C57BL/6 *Ifnar^−/−^*/*Ifngr^−/−^* mice were infected with 6.0, 5.0, 4.0, 3.0, or 2.0 log_10_ PFU/mouse of WR 7-61 and US-2003 isolates. Mice were also infected with MA-2022 at 8.0, 6.0, or 5.0 log_10_ PFU/mouse. Following infection, all mice were monitored daily for disease score, body temperature, weight loss, and survival. The disease score criteria: 0 = normal, 1 = rough coat and/or hunched posture, 20% body weight loss from baseline, 2 = mild lethargy and/or mild dyspnea, 25% body weight loss from baseline, and 3 = moderate lethargy and/or moderate dyspnea and/or 30% body weight loss from baseline. All animal dosing was verified via plaque assay and shown in respective figures.

C57BL/6 *Ifnar^−/−^* and *Ifnar^−/−^*/*Ifngr^−/−^* mice were infected via the intrarectal (IR) and intravaginal (IV) routes with WR 7-61 (7.0, 6.0, 5.0, or 4.0 log_10_ PFU) and MA-2022 (7.0 log_10_ PFU). Cohorts of mice (10 males for IR infection or 10 females for IV infection) were infected with 50 μL of virus or 1× PBS. Following infection, all mice were monitored for disease signs at the site of infection as well as the criteria above.

### Statistical analysis

Statistical significance in temperature, weight loss, and survival was evaluated using nonparametric one-way analysis of variance (ANOVA) with Tukey’s multiple comparison tests, non-parametric Mann-Whitney *t*-test, or unpaired multiple *t*-tests using a false discovery rate approach. A comparison of survival curves was performed using the log-rank (Mantel-Cox) test, the log-rank test for trend, and Gehan-Breslow-Wilcoxon tests. The representative *P*-values for each analysis are provided in the figures. All the tests were performed using GraphPad Prism software (version 10.0.1). Statistically significant *P*-values < 0.05 are shown in the graphs.

## Data Availability

The data will be made available upon request.

## SUPPLEMENTAL MATERIAL

Table S1a jvi.00247-26-s0001.xlsx Genetic polymorphisms between MPXV clade IIa isolates (US-2003 and WR 7-61) and the reference sequences.

Table S1b jvi.00247-26-s0002.xlsx Genetic polymorphisms between MPXV clade IIb isolate and the reference sequence.

ASM does not own the copyrights to Supplemental Material that may be linked to, or accessed through, an article. The authors have granted ASM a non-exclusive, world-wide license to publish the Supplemental Material files. Please contact the corresponding author directly for reuse.
